# Residual lymph node tumour burden following removal of a single axillary sentinel lymph with macrometastatic disease in women with screen-detected invasive breast cancer

**DOI:** 10.1093/bjsopen/zraa022

**Published:** 2020-12-06

**Authors:** R V Dave, S Cheung, M Sibbering, O Kearins, J Jenkins, A Gandhi

**Affiliations:** 1 The Nightingale Centre, Wythenshawe Hospital, Manchester University Hospitals NHS Foundation Trust, Manchester, UK; 2 National Health Service Breast Screening Programme, Public Health England, Birmingham, UK; 3 University Hospitals of Derby and Burton NHS Foundation Trust, Derby, UK; 4 Manchester Academic Health Sciences Centre, Faculty of Biology, Medicine and Health, University of Manchester, Manchester, UK

## Abstract

**Background:**

Women with screen-detected invasive breast cancer who have macrometastatic disease on axillary sentinel lymph node biopsy (SLNB) are usually offered either surgical axillary node clearance (ANC) or axillary radiotherapy. These treatments can lead to significant complications for patients. The aim of this study was to identify a group of patients who may not require completion ANC.

**Methods:**

Data from the NHS Breast Screening Programme between 1 April 2012 and 31 March 2017 were interrogated to identify women with invasive breast carcinoma and a single sentinel lymph node (SLN) with macrometastatic disease who subsequently proceeded to completion ANC. Univariable and multivariable analyses were performed to identify patients with a single positive SLN who had no further lymph node metastasis on ANC.

**Results:**

Of the 2401 women included in the cohort, the presence of non-sentinel node disease was significantly affected by: the number of nodes obtained at SLNB (odds ratio (OR) 0.49 for retrieval of more than 1 node), invasive size of tumour (OR 1.63 for size greater than 20 mm), surgical treatment (OR 1.34 for mastectomy), human epidermal growth factor receptor (HER) 2 status (OR 0.71 for HER2 positivity), and patient age (OR 1.10 for age less than 50 years; OR 1.46 for age greater than 70 years). Patients aged less than 70 years, with tumour size smaller than 2 cm, more than one node retrieved on SLNB, and who had breast-conserving surgery had a lower chance of positive non-sentinel nodes on completion ANC compared with other patients.

**Conclusion:**

This study, of a purely screen-detected breast cancer cohort, identified a subset of patients who may be spared completion ANC in the event of a single axillary SLN with macrometastasis.

## Introduction

Women in the UK NHS Breast Screening Programme (NHSBSP) diagnosed with invasive breast cancer and undergoing therapeutic surgery to the breast undergo preoperative assessment of their ipsilateral axillary lymph nodes by ultrasound imaging and, if appropriate, needle biopsy. If there is no proven axillary nodal disease before surgery, they will have a sentinel lymph node biopsy (SLNB) on the ipsilateral axilla, undertaken at the time of breast surgery. SLNB may reveal metastatic breast cancer in the form of isolated tumour cells (metastatic deposits of less than 2 μm in size), micrometastases (2 μm to 2 mm) or macrometastases (metastatic deposit greater than 2 mm in size). Further management of patients with isolated tumour cells or micrometastases is well established, and such patients do not require further local axillary treatment.

Ten-year outcomes of the International Breast Cancer Study Group 23-01 trial^1^ provide evidence to support the omission of axillary node clearance (ANC) in patients with tumours sized 5 cm or less who undergo breast-conserving surgery (BCS) plus adjuvant systemic therapy, and who have one or more SLNs with micrometastatic disease (2 mm or less). Patients found to have macrometastases on SLNB, in accordance with guidance from the UK National Institute of Health and Clinical Excellence (NICE)^2^ and the Association of Breast Surgery, are offered further treatment to the axilla in the form of ANC or axillary radiotherapy. Both of these treatments can result in significant complications for patients, with a 10–20 per cent risk of lymphoedema of the arm, and one-third of patients experiencing sensory change and pain in the upper arm^3^^,^^4^.

In recent years, developments in systemic therapy have raised the possibility that completion ANC following low-burden sentinel node macrometastatic disease may represent overtreatment for many patients. The ACOSOG Z0011 trial was designed to investigate this and found that, amongst patients with limited SLN metastatic breast cancer (up to 2 nodes) treated with BCS, breast radiotherapy and systemic therapy, the use of SLNB alone compared with ANC was non-inferior in terms of overall survival, disease-free survival and locoregional relapse^5^^,^^6^. Thus, completion ANC following low-burden sentinel node macrometastatic disease may represent overtreatment both in women who have no residual disease and in those who may have residual disease that can be treated effectively by systemic therapy alone. To identify the latter group, larger trials with long-term follow-up are underway that also include women undergoing mastectomy^7–9^. Recruitment to these trials is ongoing, and results will hopefully provide conclusive answers as to the best management pathway for these patients. However, the results will not be available until the mid 2020s.

Each year in the NHSBSP around 13 000 women will be diagnosed with invasive breast cancer and undergo SLNB. Approximately 1800 of these women will be found to have metastases within the SLNB and will proceed to completion ANC; half will have no further positive nodes on ANC. Multiple nomograms have been published that attempt to predict further non-sentinel node involvement using a combination of clinical and tumour pathological features^10^^,^^11^. However, these are based universally on a mixed cohort of predominantly symptomatic patients of varying ages. The applicability of these nomograms to women with screen-detected cancers is unclear, as it is recognized that the clinical behaviour of screen-detected cancers differs from that of symptomatic cancers^12^^,^^13^.

The aims of the present study were to define the number of women with screen-detected cancer with low-volume axillary disease (a single sentinel node with a macrometastatic deposit) who are found to have no further lymph node metastases on completion ANC, and to identify factors that may predict residual metastatic nodal burden upon subsequent completion axillary surgery. This could then be used to offer more informed treatment choices, such as the option to avoid completion axillary surgery after the initial finding of a single positive sentinel node in screen-detected invasive breast cancer.

## Methods

The UK NHSBSP invites women aged 50–70 years for two-view screening mammography at 3-year intervals. In addition, the programme within England randomizes some women aged 47–49 years and 71–73 years to be screened as part of the Age X (age extension) trial. Between 1 April 2012 and 31 March 2017, 12 018 962 women were screened within the NHSBSP. From this cohort, anonymized details of women who fulfilled all of the following criteria were extracted: diagnosis of invasive breast carcinoma; underwent SLNB with a single node containing macrometastatic deposits; and subsequently proceeded to completion ANC.

Patients with the following criteria were excluded from this analysis: two or more sentinel lymph nodes (SLNs) containing macrometastatic deposits; micrometastatic SLNB only; receipt of neoadjuvant systemic therapy; or diagnosis of previous breast cancer.

The NHSBSP and Association of Breast Surgery data set of screening breast cancer was interrogated to obtain the following factors: clinical data (patient age when offered screening appointment, previous breast cancer history, type of breast surgery, number of nodes obtained at SLNB, number of metastatic nodes at SLNB, residual metastatic lymph node burden at ANC); and tumour characteristics of the most aggressive breast cancer (size, grade, hormone receptor status, laterality, human epidermal growth factor receptor (HER) 2 status).

### Statistical analysis

Univariable analyses were performed using linear logistic regression to determine which clinical or pathological factors affected the outcome of a subsequent ANC (the ‘outcome’). The significance level was set at 95 per cent for the univariable analysis. Multivariable analysis was performed with the significant factors from the univariable analyses to understand the relationship between these factors and the outcome. A smaller number of factors was used for multivariable analysis in order to produce reliable results. Odds ratios (ORs) are presented, together with their 95 or 99 per cent confidence intervals. Missing data were minimal (less than 1 per cent) in this data set. Patients with missing data were excluded from analyses. Statistical analyses were performed in R software version 3.5.0 (The R Foundation for Statistical Computing, Vienna, Austria) in accordance with a prespecified statistical analysis plan. To account for multiple statistical testing within the cohort, the significance level in the multivariable analysis was set at 0.010. Interrogation of anonymized data from the NHSBSP was performed by one author, with permission granted by the Public Health England Office for Data Release and the Breast Screening Programme Research Advisory Committee.

## Results

A total of 2401 women fulfilled the inclusion criteria; 187 women (7.8 per cent) were below 50 years of age and 183 (7.6 per cent) were older than 70 years. Demographic and clinicopathological data are detailed in *Table 1*.

**Table 1:** Number and proportion of women with positive nodes found at completion axillary clearance following initial sentinel node biopsy with macrometastatic deposits in a single node

Clinical and pathological factors	No. of women (*n* = 2401)	No, with positive nodes at completion ANC
**Age at screening appointment (years)**		
<50	187	55 (29.4)
50-70	2020	554 (27.4)
>70	183	65 (35.5)
Unknown	11	
**Invasive grade**		
1	397	102 (25.7)
2	1508	428 (28.4)
3	484	145 (30.0)
Unknown	12	
**Invasive size** (mm)		
≤20	1444	350 (24.2)
>20 to ≤50	856	293 (34.2)
>50	86	32 (37)
Unknown	15	
ER status		
Negative	129	36 (27.9)
Positive	2270	642 (28.3)
Unknown	2	
**HER2 status**		
Negative	2130	618 (29.0)
Positive	236	53 (22.5)
Borderline or unknown	35	
**No. of nodes obtained at SLNB**		
1	974	359 (36.9)
≥2	1427	319 (22.4)
**Surgical treatment**		
BCS	1635	430 (26.3)
Mastectomy	766	248 (32.4)
**Laterality**		
Left	1226	342 (27.9)
Right	1175	336 (28.6)
**No. of assessment visits**		
1	2206	609 (27.6)
≥2	195	69 (35.4)

Values in parentheses are percentages. ANC, axillary node clearance; ER, oestrogen receptor; HER, human epidermal growth factor receptor; SNLB, sentinel lymph node biopsy; BCS, breast-conserving surgery.

In 974 women (40.6 per cent) a solitary SLN was obtained at SLNB (containing the macrometastases). In 718 women (29.9 per cent) two SLNs were removed (with one containing the metastases), in 596 women (24.8 per cent) three or four SLNs were removed (one of these containing metastases), in 112 women (4.7 per cent) five to nine SLNs were removed, and in one woman more than 10 nodes were removed at SLNB. Further analysis showed that the difference in residual node burden on ANC lay between one node retrieved on SLNB and multiple nodes groups, with no difference in the outcome between two, three and four or more nodes retrieved groups. Therefore, these have been combined into a single group with more than one node obtained at SLNB.

### Univariable analysis

Univariable analyses were performed to understand the relationship between the findings at subsequent ANC and each clinical or pathological factor. The outcome was defined as the presence of further positive nodes at ANC. The outcome of the subsequent ANC was significantly affected by the number of nodes obtained at SLNB (OR 0.49 for retrieval of more than 1 node), invasive size of the cancer (OR 1.63 for size greater than 20 to 50 mm), surgical treatment (OR 1.34 for mastectomy), HER2 status (OR 0.71 for HER2 positivity), and patient age (OR 1.46 for aged above 70 years) (*Table 2*). Specifically, women who had more than one node obtained at SLNB or were HER2-positive were less likely to have positive nodes at subsequent ANC. Women who were treated by mastectomy, had a larger tumour, or were aged above 70 years at the screening appointment were more likely to have positive nodes at subsequent ANC.

**Table 2:** Univariable logistic regression examining factors affecting lymph node positivity at completion axillary clearance following initial removal of a single positive sentinel node

	Odds ratio	*P*
**Age at screening appointment (years)**		
<50	1.10 (0.79, 1.52)	0.561
50–70	1.00 (reference)	
>70	1.46 (1.06, 2.00)	0.020
**No. of nodes obtained at SLNB**		
1	1.00 (reference)	
≥2	0.49 (0.41, 0.59)	<0.001
**Surgical treatment**		
BCS	1.00 (reference)	
Mastectomy	1.34 (1.11, 1.62)	0.002
**Invasive size (mm)**		
≤20	1.00 (reference)	
>20 to ≤50	1.63 (1.35, 1.96)	<0.001
>50	1.85 (1.17, 2.90)	0.008
**Invasive grade**		
1	1.00 (reference)	
2	1.15 (0.89, 1.48)	0.288
3	1.24 (0.92, 1.67)	0.161
**HER2 status**		
Negative	1.00 (reference)	
Positive	0.71 (0.51, 0.97)	0.035
**ER status**		
Negative	1.00 (reference)	
Positive	1.02 (0.69, 1.53)	0.927
**No. of assessment visits**		
1	1.00 (reference)	
≥2	1.44 (1.05,1.95)	0.0213
**Laterality**		
Left	1.00 (reference)	
Right	1.04 (0.87, 1.24)	0.703

Values in parentheses are 95 per cent confidence intervals. SNLB, sentinel lymph node biopsy; BCS, breast-conserving surgery; HER, human epidermal growth factor receptor; ER, oestrogen receptor.

### Multivariable analysis

With a significance level for the multivariable analysis at 99 per cent, two factors significantly affected the outcome of the subsequent ANC: the number of nodes obtained at SLNB and the invasive size of tumour (*Table 3*). Patients with two or more nodes obtained at SLNB were less likely to have further malignant nodes found at the subsequent completion ANC compared with patients with only one node removed (22.4 *versus* 36.9 per cent respectively) (*Table 1*) (OR 0.50, 99 per cent c.i. 0.39 to 0.64; *P* < 0.001) (*Table 3*).

**Table 3:** Multivariable logistic regression examining factors affecting lymph node positivity at completion axillary clearance following initial removal of a single positive sentinel node

	Odds ratio	*P*
**Age at first offered screening appointment (years)**		
<50	1.01 (0.63, 1.57)	0.953
50–70	1.00 (reference)	
>70	1.38 (0.89, 2.12)	0.057
**No. of nodes obtained at SLNB**		
1	1.00 (reference)	
≥2	0.50 (0.39, 0.64)	<0.001
**Surgical treatment**		
BCS	1.00 (reference)	
Mastectomy	1.24 (0.94, 1.61)	0.041
**Invasive size (mm)**		
≤20	1.00 (reference)	
>20 to ≤50	1.55 (1.20, 1.99)	<0.001
>50	1.48 (0.76, 2.81)	0.122
**HER2 status**		
Negative	1.00 (reference)	
Positive	0.69 (0.44, 1.06)	0.030
**No. of assessment visits**		
1	1.00 (reference)	
≥2	1.41 (0.92, 2.15)	0.035

Values in parentheses are 99 per cent confidence intervals. SNLB, sentinel lymph node biopsy; BCS, breast-conserving surgery; HER, human epidermal growth factor receptor.

Patients with cancers of invasive size greater than 20 to 50 mm were more likely to have positive nodes found at the subsequent completion ANC compared with those with invasive size of 20 mm or less (OR 1.55, 99 per cent c.i. 1.20 to 1.99; *P* < 0.001) (*Table 3*). Some 34.2 per cent of cancers with invasive size of more than 20 to 50 mm had positive nodes found at subsequent ANC, compared with 24.2 per cent of those with invasive size of 20 mm or less (*Table 1*).

Patients were further categorized into two groups based on the results of the univariable and multivariable analyses. The 569 women aged 70 years or less with a tumour smaller than 2 cm, more than one node retrieved on SLNB, and who had BCS were compared with the 1832 women who did not have these criteria. HER2 status was excluded due to the low number of patients with HER2 positivity. Patients who did not fulfil the above criteria had a significantly higher rate of further nodal metastasis on completion ANC (OR 1.98, 95 per cent c.i. 1.58 to 2.51; *P* < 0.001).

## Discussion

In the NHSBSP audit 2017–2018, almost 13 000 women were diagnosed with invasive breast cancer and underwent SLNB^14^. Of these, 1880 were found to have metastases within the SLNB and 773 proceeded to completion ANC. Of these, 62.9 per cent had no further metastatic axillary lymph node burden, representing possible overtreatment. In keeping with the move to de-escalate axillary surgery, many studies have been published aiming to predict those women with macrometastatic SLN disease in whom the likelihood of further axillary lymph node metastasis is so low that they can reasonably be offered omission of completion ANC surgery. These studies have been largely retrospective in nature and examined cohorts comprising of mixed symptomatic and screen-detected patients; consequently they have produced inconsistent results^11^^,^^15^^,^^16^. Several nomograms have been developed and tested, also with varying and inconsistent results^10^^,^^11^^,^^15^^,^^17^^,^^18^, mainly in mixed screening and symptomatic cohorts.

The distinction between symptomatic and screen-detected breast cancer populations is important. There is evidence that the clinical and pathological characteristics of screen-detected breast cancers are generally more favourable than those seen in symptomatic cases. The latter cancers are noted to present at a later stage, less likely to be hormone receptor-positive, to be of higher nuclear grade, and to be more likely to overexpress HER2^13,19,20^. Therefore, the currently published nomograms, based largely on symptomatic women, may not be applicable to a purely screen-detected breast cancer population.

In the present study, a group of patients who could potentially avoid completion ANC in the event of a single macrometastatic SLN was identified: women aged 70 years or less, with tumours below 20 mm in size, having BCS, and with more than one SLN removed at initial surgery. This is the largest such examination of a purely screening cohort. Prospective data capture within the NHSBSP allows for high levels of confidence in the accuracy of the data. An interesting finding of this study was that, in patients with a single macrometastatic node, retrieval of further negative SLNs was highly predictive of a negative ANC. This has been demonstrated previously^11^ in a cohort of patients from the Dutch National Cancer Registry, where the presence of additional negative SLNs resulted in more negative ANCs. Another study^21^ demonstrated that increasing the number of retrieved SLNs decreased the false-negative rate of SLNB.

The finding of a lower burden of macrometastatic non-sentinel nodes in patients with HER2-positive disease in this study is difficult to interpret, with inconsistencies in the literature. In an American series^22^, patients with triple negative disease had fewer nodal metastases than those with triple positive disease; in an Argentinian series of patients who fulfilled Z0011 trial criteria^23^, HER2 positivity was associated with a higher rate of non-sentinel node metastasis; and in an Indonesian series^24^, patients with luminal A disease had the highest rate of non-sentinel node mestastasis. There are thus inconsistencies in the influence of hormone receptor status in the development of non-sentinel node metastases.

The management of breast cancer over the decades has changed considerably from the era of the Halstedian radical mastectomy^25^. Following the publication of large randomized studies examining axillary management in patients with breast cancer there has been a clear move to de-escalate surgical intervention in the axilla. This has reduced morbidity, and trials^1^^,^^4–6^^,^^9^^,^^26^ have shown that a more conservative approach to the axilla leads to comparable survival outcomes, with reduced morbidity (in particular lymphoedema). In the USA, there has been gradual adaptation in some centres of the Z0011 trial criteria to omit completion ANC in the event of low-burden disease. The UK data from the NHSBSP national audit, which allows detailed examination of changes in clinical practice following publication of randomized trials, indicates that there has been a reluctance to follow suit, despite guidance from the Association of Breast Surgery^27^, and NICE that recommends that further axillary treatment is no longer mandatory in patients who are receiving breast conservation with whole-breast radiotherapy, who are postmenopausal, and have T1, grade 1 or 2, oestrogen receptor-positive and HER2-negative tumours^2^.

Research trials are currently underway that will provide important randomized data on optimal management of the axilla. The POSNOC trial^7^ has the potential to give ground-breaking insight into the axillary management of women with low-burden axillary SLN metastases and who are receiving systemic therapy. This trial is actively recruiting women with either screen-detected or symptomatic invasive breast cancer, and examines whether adjuvant therapy alone is non-inferior to adjuvant therapy plus axillary treatment (surgery or axillary radiotherapy) in the presence of one or two SLNs containing macrometastatic disease. It differs from the Z0011 trial in that it also includes patients treated by mastectomy, as well as those treated by BCS and breast radiotherapy. Patients undergoing mastectomy will not necessarily receive chest wall radiotherapy. The SENOMAC^28^ and SERC^8^ trials will examine a similar question. Results from these trials are not expected for many years.

## Funding information

No funding

*Disclosure*. The authors declare no conflict of interest.
